# Supplementary material for: Disentangling the influence of ecological and historical factors on seed germination and seedling types in a Neotropical dry forest
Source: PLoS One. 2020 Apr 16;15(4):e0231526. doi: 10.1371/journal.pone.0231526 (PMC7161972; doi:10.1371/journal.pone.0231526)
Supplement: S1 Table — (DOCX) [file pone.0231526.s001.docx]

**S1 Table.** Growth form of species in a tropical dry forest, in the Municipality of Churumuco, Michoacán, Mexico.

| **Family/Species** | **Growth form** |
| --- | --- |
| **Acanthaceae** |  |
| *Ruellia spissa* Leonard | Herb |
| *Justicia huacanensis* T.F. Daniel & V.W. Steinm. | Shrub |
| **Amaryllidaceae** |  |
| *Sprekelia formosissima* (L.) Herb. | Herb |
| **Anacardiaceae** |  |
| *Amphipterygium adstringens* (Schltdl.) Standl. | Tree |
| *Cyrtocarpa procera* Kunth | Tree |
| *Spondias purpurea* L. | Tree |
| **Apocynaceae** |  |
| *Marsdenia* sp. | Liana |
| *Plumeria rubra* L. | Tree |
| **Asteraceae** |  |
| *Aldama michoacana* (B.L. Turner & F.G. Davies) E.E. Schill. & Panero | Herb |
| *Bidens bigelovii* A. Gray | Herb |
| *Melampodium americanum* L. | Herb |
| *Otopappus epaleaceus* Hemsl. | Liana |
| *Pectis holochaeta* (S.F. Blake) D.J. Keil var. *holochaeta* | Herb |
| *Tagetes erecta* L. | Herb |
| *Tridax platyphylla* B.L. Rob. | Herb |
| *Zinnia americana* (Mill.) Olorode & A.M. Torres | Herb |
| *Zinnia flavicoma* (DC.) Olorode & A.M. Torres | Herb |
| **Bignoniaceae** |  |
| *Handroanthus impetiginosus* (Mart. ex DC.) Mattos | Tree |
| *Tecoma stans* (L.) Juss. ex Kunth | Shrub |
| **Bixaceae** |  |
| *Cochlospermum vitifolium* (Willd.) Spreng. | Tree |
| **Burseraceae** |  |
| *Bursera copallifera* (DC.) Bullock | Tree |
| *Bursera crenata* Paul G. Wilson | Tree |
| *Bursera fagaroides* (Kunth) Engl. | Tree |
| *Bursera grandifolia* (Schltdl.) Engl. | Tree |
| *Bursera heteresthes* Bullock | Tree |
| *Bursera infernidialis* Guevara & Rzed. | Tree |
| *Bursera sarukhanii* Guevara & Rzed. | Tree |
| **Cactaceae** |  |
| *Backebergia militaris* (Audot) Bravo ex Sánchez-Mej. | Tree |
| *Opuntia bensonii* Sánchez-Mej. | Tree |
| *Stenocereus fricii* Sánchez-Mej. | Tree |
| *Stenocereus queretaroensis* (F.A.C. Weber) Buxb. | Tree |
| *Stenocereus quevedonis* (J.G. Ortega) Buxb. | Tree |
| **Capparaceae** |  |
| *Crateva tapia* L. | Tree |
| **Caricaceae** |  |
| *Jacaratia mexicana* A. DC. | Tree |
| **Celastraceae** |  |
| *Crossopetalum managuatillo* (Loes.) Lundell | Shrub |
| **Combretaceae** |  |
| *Combretum farinosum* Kunth | Liana |
| **Convolvulaceae** |  |
| *Ipomoea* sp. | Vine |
| *Ipomoea bracteata* Cav. | Liana |
| *Ipomoea hederifolia* L. | Vine |
| *Ipomoea* sp. | Vine |
| *Ipomoea robinsonii* House | Liana |
| *Ipomoea suaveolens* (M. Martens & Galeotti) Hemsl. | Liana |
| *Ipomoea ternifolia* Cav. var. *ternifolia* | Vine |
| *Jacquemontia agrestis* (Mart. ex Choisy) Meisn. | Vine |
| *Operculina pteripes* (G. Don) O'Donell | Liana |
| **Cordiaceae** |  |
| *Cordia elaeagnoides* DC. | Tree |
| *Cordia seleriana* Fernald | Shrub |
| **Cucurbitaceae** |  |
| *Schizocarpum palmeri* Cogn. & Rose | Vine |
| **Euphobiaceae** |  |
| *Jatropha stephani* J. Jiménez Ram. & Mart. | Tree |
| *Jatropha* sp. | Tree |
| *Manihot crassisepala* Pax & K. Hoffm. | Tree |
| **Fabaceae** |  |
| *Apoplanesia paniculata* C. Presl | Tree |
| *Coulteria platyloba* (S. Watson) N. Zamora | Tree |
| *Desmodium procumbens* (Mill.) Hitchc. var. *exiguum* (A. Gray) B.G. Schub. | Herb |
| *Erythrina oliviae* Krukoff | Tree |
| *Erythrostemon macvaughii* (J.L. Contr. & G.P. Lewis) Gagnon & G.P. Lewis | Tree |
| *Galactia acapulcensis* Rose | Liana |
| *Galactia viridiflora* (Rose) Standl. | Liana |
| *Haematoxylum brasiletto* H. Karst. | Tree |
| *Heteroflorum sclerocarpum* M. Sousa | Tree |
| *Libidibia coriaria* (Jacq.) Schltdl. | Tree |
| *Lonchocarpus longipedunculatus* M. Sousa & J. C. Soto | Tree |
| *Lonchocarpus schubertiae* M. Sousa | Tree |
| *Lysiloma divaricatum* (Jacq.) J.F. Macbr. | Tree |
| *Lysiloma tergeminum* Benth. | Tree |
| *Mariosousa acatlensis* (Benth.) Seigler & Ebinger | Tree |
| *Mimosa albida* Humb. & Bonpl. ex Willd. | Shrub |
| *Mimosa arenosa* (Willd.) Poir. | Tree |
| *Mimosa rosei* B.L. Rob. | Shrub |
| *Mimosa* sp. | Tree |
| *Parkinsonia praecox* (Ruiz & Pav. ex Hook.) Hawkins | Tree |
| *Piptadenia flava* (Spreng. ex DC.) Benth. | Tree |
| *Piptadenia obliqua* (Pers.) J.F. Macbr. | Tree |
| *Pithecellobium oblongum* Benth. | Shrub |
| *Poincianella eriostachys* (Benth.) Britton & Rose | Tree |
| *Prosopis laevigata* (Humb. & Bonpl. ex Willd.) M.C. Johnst. | Tree |
| *Pterocarpus orbiculatus* DC. | Tree |
| *Ramirezella strobilophora* (B.L. Rob. ex Pringle) Rose | Vine |
| *Senegalia picachensis* (Brandegee) Britton & Rose | Tree |
| *Senna obtusifolia* (L.) H.S. Irwin & Barneby | Vine |
| *Senna uniflora* (Mill.) H.S. Irwin & Barneby | Herb |
| *Senna wislizeni* (A. Gray) H.S. Irwin & Barneby | Shrub |
| *Zapoteca formosa* (Kunth) H.M. Hern. subsp. *rosei* (Wiggins) H.M. Hern. | Shrub |
| **Lamiaceae** |  |
| *Hyptis suaveolens* (L.) Poit. | Herb |
| *Salvia uruapana* Fernald | Herb |
| **Lythraceae** |  |
| *Cuphea leptopoda* Hemsl. | Herb |
| **Malpighiaceae** |  |
| *Malpighia mexicana* A. Juss. | Tree |
| **Malvaceae** |  |
| *Anoda palmata* Fryxell | Herb |
| *Ayenia micrantha* Standl. | Shrub |
| *Ayenia purpusii* Brandegee | Herb |
| *Ceiba aesculifolia* (Kunth) Britten & Baker f. | Tree |
| *Gossypium lobatum* Gentry | Shrub |
| *Heliocarpus occidentalis* Rose | Tree |
| *Melochia tomentosa* L. | Shrub |
| *Sida glabra* Mill. | Herb |
| **Nyctaginaceae** |  |
| *Salpianthus standleyi* Steyerm. | Shrub |
| **Plocospermataceae** |  |
| *Plocosperma buxifolium* Benth. | Shrub |
| **Polygonaceae** |  |
| *Ruprechtia fusca* Fernald | Tree |
| **Resedaceae** |  |
| *Forchhammeria pallida* Liebm. | Tree |
| **Rhamnaceae** |  |
| *Karwinskia johnstonii* R. Fernández | Tree |
| **Rubiaceae** |  |
| *Genipa americana* L. | Tree |
| *Guettarda elliptica* Sw. | Tree |
| *Randia thurberi* S. Watson | Shrub |
| **Solanaceae** |  |
| *Datura kymatocarpa* A.S. Barclay | Herb |
| *Physalis* sp. | Herb |
| *Solanum grayi* Rose | Herb |
| **Verbenaceae** |  |
| *Lantana hirta* (Cham.) Meisn. ex Walp. | Shrub |
| **Vitaceae** |  |
| *Ampelocissus acapulcensis* (Kunth) Planch. | Liana |
| *Cissus tiliacea* Kunth | Liana |
| *Cissus verticillata* (L.) Nicolson & C.E. Jarvis | Liana |
