# Supplementary material for: Disentangling the influence of ecological and historical factors on seed germination and seedling types in a Neotropical dry forest
Source: PLoS One. 2020 Apr 16;15(4):e0231526. doi: 10.1371/journal.pone.0231526 (PMC7161972; doi:10.1371/journal.pone.0231526)
Supplement: S2 Table — GF: growth form, DS: dispersal syndrome, DP: seed dispersal phenology, SM: seed mass, AIC: Akaike information criterion, Δi: delta Akaike. (DOCX) [file pone.0231526.s002.docx]

**S2 Table.** Selection of the phylogenetic generalized least squares models constructed to evaluate the correlations between attributes, based on the Akaike information criterion. GF: growth form, DS: dispersal syndrome, DP: seed dispersal phenology, SM: seed mass, AIC: Akaike information criterion, Δi: delta Akaike.

| **Model** | **D.F.** | **AIC** | **Δi** |
| --- | --- | --- | --- |
| GF*SM + DS*SM + DP*SM | 20 | 137.99 | 3.27 |
| GF + DS + DP + SM | 11 | 140.66 | 5.94 |
| DS + GF/SM | 8 | 134.72 | 0 |
| GF + DS | 7 | 135.44 | 0.72 |
| Germination rate |  |  |  |
| GF*SM + DS*SM + DP*SM | 20 | 159.34 | 14.82 |
| GF + DS + DP + SM | 11 | 151.46 | 6.94 |
| GF/SM + DS/SM | 8 | 144.51 | 0 |
| GF + DS + SM | 8 | 151.17 | 6.66 |
