# Supplementary figures and images for: Disentangling the influence of ecological and historical factors on seed germination and seedling types in a Neotropical dry forest
Source: PLoS One. 2020 Apr 16;15(4):e0231526. doi: 10.1371/journal.pone.0231526 (PMC7161972; doi:10.1371/journal.pone.0231526)

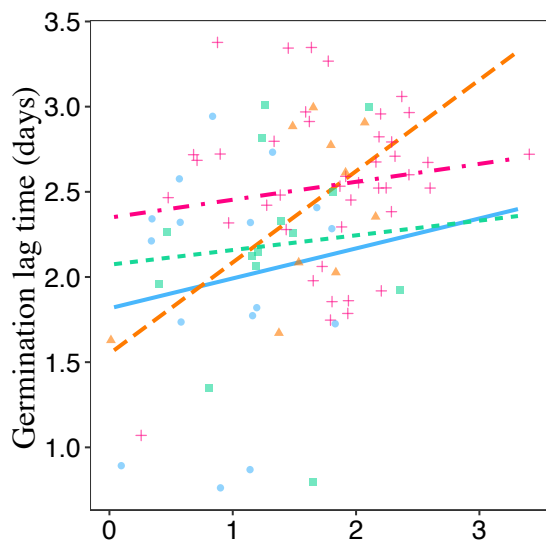

A

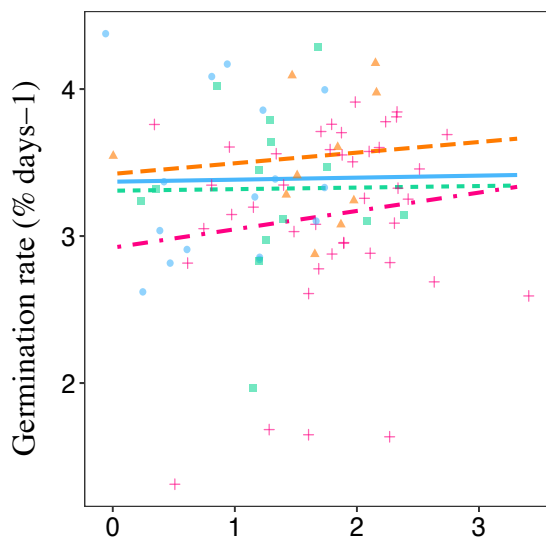

B

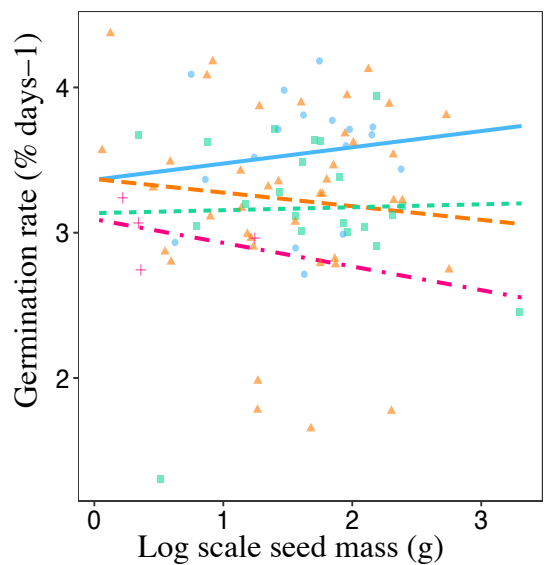

Anemochory  
Autochory  
Endozoochory  
Epizoochory

Supplement: S1 Fig — Interaction of growth form and dispersal syndrome with seed mass to explain the variation in the lag time germination (A), and the germination rate (B). (PDF) [file pone.0231526.s003.pdf]
