# Supplementary material for: Disentangling the influence of ecological and historical factors on seed germination and seedling types in a Neotropical dry forest
Source: PLoS One. 2020 Apr 16;15(4):e0231526. doi: 10.1371/journal.pone.0231526 (PMC7161972; doi:10.1371/journal.pone.0231526)
Supplement: Attachment — Submitted filename: renamed_4e552.docx [file pone.0231526.s004.docx]

**Reviewer #1**

This paper looks to be much improved. The figures have been improved. The organization is much better, and the language and focus have been polished to where it much more understandable. Most all of the comments were incorporated in the manuscript to a satisfactory degree. I commend the authors on thorough revision.

**Comment 1:** L 33: which mostly associate

**Response 1:** We made the change suggested (lines 33-34).

**Reviewer #4**

In this study, the authors examine the correlates of germination success in a tropical dry forest. Specifically, they examine how seed germinate rates and lag time can be explained by species’ seed dispersal phenology, seed traits, dispersal mode and evolutionary history. Early life history dynamics such as seed-to-seedling transition plays a key part in plant community ecology and this study adds to that body of knowledge. Moreover, as best as I know, most seed/seedling studies in forests have been done in wetter regions, so this study from dry forests can increase the representation of biomes in the literature on early life-stage dynamics of plants. The inclusion of different life-forms is also valuable. However, I find that some of the arguments for expected relationships are not intuitively clear in terms of the biological significance.

**Comment 1.** L: 93-94, are you saying that despite differences in phenology, species will germinate at the same time or that most plants would disperse seeds at end of dry season?

**Response 1.** We rewrite this sentence and explained that seed germination would be more frequent in species that disperse their seeds at the end of the dry or beginning of the rainy season (lines 94-96).

**Comment 2.** Similarly, the link between dispersal syndrome, seed mass and lag time (line 99-100) needs more detail. It is also not entirely clear to me why seedling types matter. Please explain the ecological significance of understanding differences in seedling types and their correlation with life-history traits.

**Response 2.** We agree with these valuable comments and in the current version we specified that since lag time and rate of germination can vary with the thickness of the seed coat, we expected that germination to be faster in seeds with a thin coat of wind-dispersed species, than in seeds with a thick coat of animals-dispersed species (lines 100-101). Besides, we added that the functional morphology of cotyledons (seedling types) is important on seedling establishment because seedling types are associated with other plant life-history traits. Particularly, we predicted that seedlings with foliaceous cotyledons would be more frequent in herbs (they have predominantly small seeds, therefore the lag time and germination rate would be faster), whereas seedlings with reserve cotyledons would be more frequent in woody species (associated more often with large seeds and, therefore, the lag time and germination rate would be slower). (lines 101-108).

**Comment 3**. The introduction ends with what was done rather than why it was done. It would help the reader to have your objectives written out as questions or more investigative statements. I suggest distilling the arguments from the previous paragraphs into statements of the specific research questions or gaps that you addressed here. For instance, can you provide a one-line justification for why you characterized dispersal phenology, dispersal syndromes etc. In general, the introduction can be reorganized to present specific packets of information in the order that they lead up to the main questions so as to avoid being repetitive and have better flow.

**Response 3**. The reviewer’ comments are correct. In this modified version we write our objectives as questions (lines 90-93).

**Comment 4.** The methods for shadehouse experiments need to be explained more clearly. Ten seeds per bag seem like a lot for the size of the bag. Did you collect all local soil from the same location?

**Response 4.** We did not have a problem with the size of the bag, because we use black polythene bags of 20 × 15 × 30 cm, i.e., we sowed ten seeds in an area of 300 cm^2^. In relation with the second question, we added in the manuscript that soil was collected exclusively from the same place (lines 167-168).

**Comment 5.** Did you apply any pesticide treatment to the soil to avoid enemy infestation? If you do not control for insects or fungal infection, please discuss whether enemies might have differentially affected the germination of species with different traits (e.g., seed size, seed coat thickness).

**Response 5.** We specified now that in order to germinate the seeds in the most similar conditions to occurs naturally, which was one of the objectives to developt in this research, we do not apply any pesticides or fertilizers to the soil (lines 169-170). We had not any problems in the seed germination experiments related with pests or predators and thus, we do not mention nothing of this topic in the manuscript.

**Comment 6.** As pointed out by a previous reviewer, the models need to be better explained/described. Until the results, the reader does not know how many models have been run, how they were set up (response and predictors) and how was model performance/fit assessed and models compared. Clearly, you had a multiple models per response, but how were they different?

**Response 6.** We thank the reviewer for this comment. In lines 203-204 of the data analysis section, we mentioned that model selection was based on the Akaike information criterion (AIC). In the S2 Table, we describe all the models tested and show that the model selected was the one with the lowest delta AIC (i.e., the model with the best fit). In the heading of the Table 1, we show the final model and the results and indicate that lag time and rate of germination are the response variables (line 269).

**Comment 7.** Did you conduct post-hoc pairwise tests for the different categorical variables (corrected for multiple comparisons)?

**Response 7.** We do not conduct a post-hoc pairwise test for the categorical variables. For each level of categorical variables, we reported the estimated coefficients (β) and the p-value of the pgls models tested.

**Comment 8.** I am not sure whether you tested for interactions among variables. I would like to see whether the patterns for seed mass and germination rates were driven by life-form or were the slopes similar within each life-form. Does the forward slash in Table 1 indicate an interaction? Why does the second model not have the main effects of the variables? It would be helpful to see plots and predicted lines for some of the relationships tested. The paper seems a bit heavy on tables and lean on figures.

**Response 8.** All these comments are interesting. First, we do test the interactions among the predictors. For example, the best fit model to explain germination lag time included dispersal syndrome and growth form in interaction with seed mass as predictors. In the second model, the germination rate was explained by dispersal syndrome and growth form, both in interaction with seed mass. Second, in Table 1, we added that forward slash indicates the interactions between traits (we indicate this relation in the lines 270-271). Third, we believe that Table 1 shows clearly and synthetically the effects of explicative variables on response variables. A acknowledge that a figure can be visually useful, but the statistics values cannot be displayed completely. To resolve this controversy, we made the figure suggested by the reviewer and it will appear as supplementary material in the current version of the manuscript.

**Comment 9**. Line 42: it is not clear why seedling establishment is based on resource acquisition for all species. What about seed resources?

**Response 9.** In order to be clearer, in the current version we specified that successful seedling establishment depends on the seed resources and rapid acquisition of available environmental resources (line-42).

**Comment 10.** Line 67: you mean rates of emergence?

**Response 10.** Thanks for this observation; we inserted the word emergence (line 67).

**Comment 11.** Line 85: can you briefly justify why studies from trees may not be generalizable to herbs?

**Response 11.** We did it. In lines 83-84 we specified that trees have life-history strategies different from other growth forms supported by three references.

**Comment 12.** Line 95: what is the difference between rate and percentage?

**Response 12.** Because germination rate is calculated from the slope at the inflection point of the curve of germination, this metric includes percentage plus velocity of germination.

**Comment 13.** Line 99: needs citation: “thickness of the seed coat can vary with dispersal syndrome”

**Response 13.** We made the change suggested (inserted reference 11).

**Comment 14.** Line 136: briefly explain how the statistical procedure works

**Response 14.** We thank the reviewer for this suggestion. In lines 141-143, we explained that to calculate the mean angle (ā), months were converted into angles (e.g., January corresponds to 15°, February to 45° and so, and that the statistical significance of ā was determined using Rayleigh’s test (z).

**Comment 15.** Line 138: ‘mature fruit’?

**Response 15.** This observation is correct; we made the change suggested (line 139).

**Comment 16.** Line 162: what was the rationale for burying seeds this way?

**Response 16.** In lines 172-174 we mentioned that each seed was buried at a depth of approximately twice the seed width to prevent dessication of seed and besides, to facilitate its emergence.

**Comment 17.** Line 169: I am a bit confused about this. Did you dig up the seeds or use a fresh set that was scarified?

**Response 17.** We thank the reviewer for this comment. In lines 179-180, we specified that pregerminative treatments were applied to a new set of the seed of those species in which did not occur germination.

**Comment 18.** Line 170: why then did you scarify seeds? What was that used for?

**Response 18.** In the methods, we separated into two sections the seed germination experiment and the seedling types characterization. This experiment allowed us to identify which species not germinate and so, require some treatment to induce the germination and obtain the seedling (lines 179-181).

**Comment 19.** Line 197: what assumptions were those?

**Response 19.** We acknowdledge the reviewer's comment. We rewrote the text in lines 207-208: the germination percentage data were arcsine transformed to be included in the analyzed models.

**Comment 20.** Lines 203-213: why were predictors used separately?

**Response 20.** We related predictors separately because they are categorical variables, and the phylogenetic logistic regression analysis cannot form interactions between these predictors. Therefore, in Table 2, we report the coefficients for all levels of categorical predictors.
